# Supplementary material for: AXIN1 in Plasma or Serum Is a Potential New Biomarker for Endometriosis
Source: Int J Mol Sci. 2019 Jan 7;20(1):189. doi: 10.3390/ijms20010189 (PMC6337238; doi:10.3390/ijms20010189)
Supplement: Supplementary file 1 [file ijms-20-00189-s001.pdf]

Supplementary Materials

AXIN1 in Plasma or Serum Is a Potential New Biomarker for Endometriosis

Table S1. Inflammation-related proteins measured by Proximity Extension Assay.

**Protein**	**Abbreviation**
Interleukin-8	IL-8
Vascular endothelial growth factor A	VEGF-A
Brain-derived neurotrophic factor	BDNF
Monocyte chemotactic protein 3	MCP-3
Glial cell line-derived neurotrophic factor	hGDNF
CUB domain-containing protein 1	CDCP1
Natural killer cell receptor 2B4	CD244
Interleukin-7	IL-7
Osteoprotegerin	OPG
Latency-associated peptide transforming growth factor beta 1	LAP TGF-beta-1
Urokinase-type plasminogen activator	uPA
Interleukin-6	IL-6
Interleukin-17C	IL-17C
Monocyte chemotactic protein 1	MCP-1
Interleukin-17A	IL-17A
C-X-C motif chemokine 11	CXCL11
Axin-1	AXIN1
TNF-related apoptosis-inducing ligand	TRAIL
Interleukin-20 receptor subunit alpha	IL-20RA
C-X-C motif chemokine 9	CXCL9
Cystatin D	CST5
Interleukin-2 receptor subunit beta	IL-2RB
Interleukin-1 alpha	IL-1 alpha
Oncostatin-M	OSM
Interleukin-2	IL-2
C-X-C motif chemokine 1	CXCL1
Thymic stromal lymphopoietin	TSLP
C-C motif chemokine 4	CCL4
T cell surface glycoprotein CD6 isoform	CD6
Stem cell factor	SCF
Interleukin-18	IL-18
Signaling lymphocytic activation molecule	SLAMF1
Transforming growth factor alpha	TGF-alpha
Monocyte chemotactic protein 4	MCP-4
Eotaxin-1	CCL11
Tumor necrosis factor ligand superfamily member 14	TNFSF14
Fibroblast growth factor 23	FGF-23
Interleukin-10 receptor subunit alpha	IL-10RA

Fibroblast growth factor 5	FGF-5
Matrix metalloproteinase-1	MMP-1
Leukemia inhibitory factor receptor	LIF-R
Fibroblast growth factor 21	FGF-21
C-C motif chemokine 19	CCL19
Interleukin-15 receptor subunit alpha	IL-15RA
Interleukin-10 receptor subunit beta	IL-10RB
Interleukin-22 receptor subunit alpha-1	IL-22 RA1
Interleukin-18 receptor 1	IL-18R1
Programmed cell death 1 ligand 1	PD-L1
Beta-nerve growth factor	Beta-NGF
C-X-C motif chemokine 5	CXCL5
TNF-related activation-induced cytokine	TRANCE
Hepatocyte growth factor	HGF
Interleukin-12 subunit beta	IL-12B
Interleukin-24	IL-24
Interleukin-13	IL-13
Artemin	ARTN
Matrix metalloproteinase-10	MMP-10
Interleukin-10	IL-10
Tumor necrosis factor	TNF
C-C motif chemokine 23	CCL23
T-cell surface glycoprotein CD5	CD5
Macrophage inflammatory protein 1-alpha	MIP-1 alpha
Fms-related tyrosine kinase 3 ligand	Flt3L
C-X-C motif chemokine 6	CXCL6
C-X-C motif chemokine 10	CXCL10
Eukaryotic translation initiation factor 4E-binding protein 1	4E-BP1
Interleukin-20	IL-20
SIR2-like protein 2	SIRT2
C-C motif chemokine 28	CCL28
Delta and Notch-like epidermal growth factor-related receptor	DNER
Protein S100-A12	EN-RAGE
CD40L receptor	CD40
Interleukin-33	IL-33
Interferon gamma	IFN-gamma
Fibroblast growth factor 19	FGF-19
Interleukin-4	IL-4
Leukemia inhibitory factor	LIF
Neurturin	NRTN
Monocyte chemotactic protein 2	MCP-2
Caspase 8	CASP-8
C-C motif chemokine 25	CCL25
Fractalkine	CX3CL1
Tumor necrosis factor receptor superfamily member 9	TNFRSF9

Neurotrophin-3	NT-3
Tumor necrosis factor (Ligand) superfamily, member 12	TWEAK
C-C motif chemokine 20	CCL20
Sulfotransferase 1A1	ST1A1
STAM-binding protein	STAMPB
Interleukin-5	IL-5
Adenosine Deaminase	ADA
TNF-beta	TNFB
Macrophage colony-stimulating factor 1	CSF-1

Table S2. Excluded proteins in Proximity Extension Assay.

Protein	Abbreviation	Reason for Exclusion
Artemin	ARTN	>20% of values below LOD
Brain-derived neurotrophic factor	BDNF	>20% of values below LOD
Interferon gamma	IFN-γ	>20% of values below LOD
Interleukin-1 alpha	IL-1 alpha	>20% of values below LOD
Interleukin-2	IL-2	>20% of values below LOD
Interleukin-2 receptor subunit beta	IL-2RB	>20% of values below LOD
Interleukin-4	IL-4	>20% of values below LOD
Interleukin-5	IL-5	>20% of values below LOD
Interleukin-8	IL-8	Technical difficulties
Interleukin-10 receptor subunit	IL-10RA	
Interleukin-13	IL-13	>20% of values below LOD
Interleukin-17A	IL-17A	>20% of values below LOD
Interleukin-17C	IL-17C	>20% of values below LOD
Interleukin-20	!L-20	>20% of values below LOD
Interleukin-20 receptor subunit alpha	IL-20 RA	>20% of values below LOD
Interleukin-22 receptor subunit alpha-1	IL-22 RA1	>20% of values below LOD
Interleukin-24	IL-24	>20% of values below LOD
Interleukin-33	IL-33	>20% of values below LOD
Leukemia inhibitory factor	LIF	>20% of values below LOD
Monocyte chemotactic protein 3	MCP-3	>20% of values below LOD
Neurtuin	NRTN	>20% of values below LOD
Thymic stromal lymphopoietin	TSLP	>20% of values below LOD
Tumor necrosis factor	TNF	>20% of values below LOD

Table S3. Principal component analysis of inflammation-related proteins.

Protein	Factor 1	Factor 2	Factor 3	Factor 4
AXIN1	0.239	0.886	0.002	0.159
SIRT2	0.349	0.869	0.067	0.181
STAMBP	0.375	0.863	0.059	0.107
ST1A1	0.215	0.821	0.009	0.173
CASP8	0.462	0.594	0.380	0.158
IL-7	0.493	0.563	0.282	-0.291
CXCL1	0.468	0.449	0.043	-0.500
CXCL5	0.617	0.434	-0.023	-0.409
CXCL6	0.637	0.431	0.055	-0.323
CD40	0.804	0.422	-0.113	0.051
TNFSF14	0.660	0.411	0.287	0.072
E4BP1	0.783	0.392	0.109	-0.067
NT3	0.268	0.390	-0.148	0.345
TGFbeta1	0.863	0.322	-0.013	-0.132
ENRANGE	0.091	0.269	-0.026	0.080
CD244	0.827	0.258	-0.149	0.159
CXCL11	0.692	0.240	0.297	-0.236
ADA	0.721	0.217	-0.051	0.054
PDL1	0.674	0.093	0.130	0.303
MCP2	0.791	0.044	-0.051	-0.176
IL-18	0.750	0.033	0.100	0.082
OSM	0.359	0.031	0.123	0.036
MCP4	0.617	0.030	0.393	-0.385
TNFB	0.522	0.022	-0.100	0.240
MMP1	0.651	0.020	0.156	-0.324
β-NGF	0.296	0.019	0.148	0.174
TRANCE	0.440	0.012	-0.157	0.121
VEGFA	0.898	-0.003	-0.096	-0.178
FGF23	0.537	-0.005	0.121	0.118
CSF1	0.881	-0.008	-0.313	0.075
DNER	0.846	-0.020	-0.365	-0.033
GDNF	0.435	-0.027	0.180	0.097
SCF	0.764	-0.043	-0.4112	-0.042
CCL23	0.839	-0.051	-0.285	0.000
TWEAK	0.862	-0.057	-0.368	-0.100
CCL19	0.717	-0.070	-0.003	0.115
IL-18RI	0.817	-0.072	-0.111	0.160
IL-6	0.466	-0.082	0.351	0.108
uPA	0.883	-0.087	-0.313	-0.094
IL-10RB	0.865	-0.091	-0.186	0.215

CST5	0.731	-0.120	-0.190	-0.096
IL12B	0.642	-0.120	-0.026	0.389
TRAIL	0.887	-0.126	-0.239	-0.041
HGF	0.895	-0.133	0.008	-0.077
CCL4	0.758	-0.136	0.134	-0.040
CD5	0.833	-0.136	-0.085	0.311
CX3CL1	0.802	-0.137	-0.178	0.131
CD6	0.727	-0.139	-0.079	0.191
OPG	0.873	-0.160	-0.229	-0.090
FGF19	0.475	-0.164	-0.341	0.016
TGF-α	0.761	-0.165	0.052	0.158
FIt3I	0.829	-0.177	-0.144	-0.057
LIFR	0.570	-0.180	0.013	0.226
CCL28	0.387	-0.193	0.319	-0.006
MIP1-α	0.562	-0.196	0.418	0.127
MMP-10	0.680	-0.204	-0.058	-0.249
SLAMF1	0.535	-0.204	0.293	0.218
FGF21	0.352	-0.206	0.362	0.018
CCL20	0.498	-0.208	0.082	0.096
FGF5	0.401	-0.217	0.367	0.061
IL-15RA	0.509	-0.218	0.433	0.363
MCP-1	0.849	-0.234	-0.080	-0.209
CXCL10	0.678	-0.266	0.121	-0.061
CCL25	0.707	-0.279	0.026	-0.196
TNFRSF9	0.770	-0.289	0.113	0.153
IL-10	0.507	-0.345	0.222	0.231
CCL11	0.763	-0.363	0.069	-0.306
CDCP1	0.483	-0.369	0.443	-0.144
CXCL9	0.657	-0.401	0.314	-0.201

Table S4. Gastrointestinal symptoms in microscopic colitis and protein levels.

Protein	Abdominal Pain	Diarrhea	Constipation	Bloating and Flatulence	Vomiting and Nausea
MCP3	R = 0.329<br>P = 0.024	R = 0.317<br>P = 0.032			
CD6					R = 0.308<br>P = 0.035
CCL11		R = 0.360<br>P = 0.017			
LIF-R		R =-0.346<br>P = 0.019			
IL-18R1	R = 0.366<br>P = 0.011				R = 0.294<br>P = 0.045
CXCL5				R = -0.318<br>P = 0.029	
MIP-1 alpha					
CCL25	R = 0.297<br>P = 0.042				
NT-3		R = -0.366<br>P = 0.012			
CSF 1					R = 0.308<br>P = 0.035

Spearman´s correlation test. *p*-values < 0.1 were presented. *p*-values < 0.01 were considered statistically significant.
